# Intention of Parents to Immunize Children against SARS-CoV-2 in Italy

**DOI:** 10.3390/vaccines9121469

**Published:** 2021-12-11

**Authors:** Luisa Russo, Ileana Croci, Ilaria Campagna, Elisabetta Pandolfi, Alberto Villani, Antonino Reale, Maria Antonietta Barbieri, Massimiliano Raponi, Francesco Gesualdo, Alberto Eugenio Tozzi

**Affiliations:** 1 Multifactorial and Complex Diseases Research Area, Bambino Gesù Children’s Hospital, IRCCS, 00165 Rome, Italy; luisa.russo@opbg.net (L.R.); ileana.croci@opbg.net (I.C.); ilaria.campagna@opbg.net (I.C.); elisabetta.pandolfi@opbg.net (E.P.); albertoeugenio.tozzi@opbg.net (A.E.T.); 2 Pediatric Emergency Department, Bambino Gesù Children’s Hospital, IRCCS, 00165 Rome, Italy; alberto.villani@opbg.net (A.V.); antonino.reale@opbg.net (A.R.); mantonietta.barbieri@opbg.net (M.A.B.); 3 Health Direction, Bambino Gesù Children’s Hospital, IRCCS, 00165 Rome, Italy; massimiliano.raponi@opbg.net

**Keywords:** COVID-19, vaccines, children, vaccine hesitancy

## Abstract

Several countries have targeted adolescents for immunization against SARS-CoV-2 to mitigate COVID-19 spread. In Italy, immunization for children ≥ 12 years has been available starting from June 2021. We conducted a cross-sectional study to investigate the knowledge, attitude and intention to vaccinate children < 18 years in Italian families. We used a multinomial logistic regression model to investigate factors associated with intention to vaccinate. We collected a total of 1696 responses. Among the 491 families of children ≥ 12 years, 41.2% would not vaccinate their children and 21.2% were uncertain, while among the 1205 families of children < 12 years, 36.1% would not vaccinate and 33.8% were uncertain. Determinants of intention to vaccinate both age groups were perceived safety and efficacy of vaccines and perceived risk of transmitting infection to adults. For children < 12 years, additional determinants were perceived risk of being infected and being hospitalized because of COVID-19. In view of the expanding strategy to vaccinate adolescents and the availability of immunization for children < 12 years, our results call for a communication strategy targeted at families of children focused on the safety and efficacy of COVID-19 vaccine in children and on the dynamics of infection spread across different age groups. As perceptions in families are volatile and may change rapidly over time, repeated surveys for measuring attitudes to vaccinate would be advisable.

## 1. Introduction

The World Health Organization estimates that almost 230 million cases of COVID-19 have occurred worldwide as of September 2021, with more than 4.5 million deaths [1]. The first cases of COVID-19 in Europe were recorded in January 2020, and, on 31 January, two Chinese tourists were reported as the first two COVID-19 cases in Italy [2]. Since then, Italy has been severely hit by the pandemic [3].

COVID-19 severity and mortality increases with age, with the highest figures seen in >85 years and in younger individuals with co-morbidities [4]. In children, the COVID-19 clinical picture is usually mild [5], requiring only supporting care, and death is rare. A recent meta-analysis [6] showed that >90% of children younger than 5 years with a laboratory-confirmed infection have a mild to moderate disease. Nevertheless, severe cases may occur, with a reported frequency of 1 to 8% [7], and 25% of severe cases in children may require ICU admission. Patients younger than 1 year of age and children with co-morbidities are at a higher risk of developing a severe illness and being admitted to the ICU [8].

During the first phase of the pandemic in Italy, incidence rates were higher in adults and in the elderly. After a severe restriction in social contacts from March 2020, including school attendance, travels and social gatherings, limitations were gradually lifted in summer 2020 [9]. Starting from August 2020, the median age of cases decreased sharply, indicating an increased incidence rate in younger cohorts, and their potential role as a source of infections for other individuals in the household [10]. After the summer season, the number of new cases increased again as the median age of cases did. During the 2020/2021 season, school attendance for children was limited as COVID-19 figures increased, on the basis of a regional “traffic-light” system which modulated restrictions in different Italian regions based on epidemiological indicators. After July 2021, an increased circulation of the SARS-CoV2 Delta variant possibly caused a more sustained circulation of the infection in children and young people in 2021 [11,12].

Immunization of the elderly and adults started in Italy at the beginning of 2021, while COVID-19 vaccines have been actively offered to teenagers 12–18 years since June 2021. Although the role of children in sustaining the circulation of SARS-CoV2 seems limited [13,14], immunization of children aims to limit the circulation of the virus through increasing the number of immune individuals, to prevent secondary infections in the household, and to gradually restore school attendance and other social activities in this age group. For this reason, the start of the immunization campaign for children 12–18 years old was set before school opening.

Monitoring intention to immunize children may be important to predict immunization rates in this age group and in children less than 12 years old who will possibly be shortly targeted by the immunization campaign [15].

Variable levels of vaccine hesitancy for COVID-19 childhood immunization have been observed in different countries, including the US and Italy [16,17], at different times. Moreover, in 2020, a survey conducted in families of Italian children showed low levels of intention to vaccinate children in a population generally favorable to pediatric vaccinations [18].

An attempt to monitor vaccine intention over time has been developed by Johns Hopkins Center for Communication Programs in collaboration with Facebook, which is updated every two weeks [19]. This information source suggests that in Italy, in August 2021, nearly 73% and 88% of respondents stated that they would vaccinate their children in primary school or below and those in high school, respectively [19].

On the other hand, no surveys have been conducted recently to gather information on vaccine intention and vaccine confidence toward COVID-19 children’s immunization and their determinants, since vaccines became approved in Italy for children older than 11 years.

For these reasons, in July and August 2021 we conducted a survey on parents of Italian children < 18 years old to investigate their intention to vaccinate their children and the factors associated with their attitude.

## 2. Materials and Methods

This is a cross-sectional study, conducted in July and August 2021, investigating intention to immunize their children and confidence in the COVID-19 vaccine in a population of parents of children < 18 years. Parents accessing the Emergency Room of the Bambino Gesù Children’s Hospital, IRCCS (Istituto di Ricerca e Cura a Carattere Scientifico, Scientific Institute for Research, Hospitalization and Healthcare), were invited to fill in an online, anonymous survey via SurveyMonkey^®^ (San Mateo, CA, USA), after providing informed consent online. Bambino Gesù Children’s Hospital is a 607-bed tertiary care academic hospital located in the Lazio Region, Italy. It is one of the largest pediatric hospitals in Europe, with more than 2 M healthcare services provided yearly to a wide population of children and adolescents. The survey was active from 22 July to 31 August 2021. Participants were asked to promote the study through their contacts, including through social networks. The only inclusion criterion was having at least one child younger than 18 years of age.

This study was approved by the Ethics Committee of the Bambino Gesù Children’s Hospital in Rome (protocol 1059).

### 2.1. Questionnaire

Data were collected using a structured questionnaire, which included 21 items divided into 4 sections.

In the first section, socio-demographic and other general information were investigated through the following items: age; sex; region; study title; number of household members (including the respondent); number of children; age and school attendance for each child; presence in the household of individuals affected by one of the underlying diseases for which the COVID-19 vaccination is recommended; having received the influenza vaccine in the 2020/2021 season; respondents and/or other household member’s history of COVID-19 (including hospitalization); having received a vaccine against COVID-19 (possible answers: yes; no I won’t get the vaccine; no but I’ll do it as soon as it is available for my age; no as I have had COVID-19 less than 3 months ago; no but I’ve booked the vaccination; I am not sure).

The second section investigated perception of COVID-19 and COVID-19 vaccination: perceived risk of getting COVID-19 in children < 18 years; perceived risk of COVID-19 hospitalization in children < 18 years; perceived risk of children affected by COVID-19 transmitting the virus compared to adults; perceived effectiveness of COVID-19 vaccines; perceived risks of COVID-19 vaccines (with more than one answer allowed: vaccines do not cause major side effects; vaccines frequently cause mild side effects like fever and tiredness; vaccines can rarely cause severe adverse events; vaccines are dangerous; I do not know).

The third section investigated attitude towards children’s COVID-19 vaccination, i.e., intention to vaccinate children when vaccine is available for their age range (yes I will; they have already been vaccinated; no; I do not know). Those who declared that they would vaccinate or that had already vaccinated their child were asked to provide an explanation of their choice from the following (multiple answers were allowed): vaccine against COVID-19 is the quickest way to have my child back to normal life; it will protect my child from COVID-19 and its complications; it will contribute to preventing COVID-19 in our family; it will contribute to herd immunity; I unconditionally trust vaccines. Those who declared that they would not vaccinate their child or that they were undecided were asked to provide an explanation of their choice from the following (multiple answers were allowed): I have fear of side effects; vaccines have not been studied enough in children; the risk of getting COVID-19 in children is low; the risk of developing COVID-19 complications in children is low; the COVID-19 vaccine has a low efficacy; I don’t trust vaccines in general; I do not know.

### 2.2. Statistical Analysis

The sample size for the survey was calculated according to the formula adopted in the Raosoft software [20]. Setting the expected proportion of the outcome found in each question of the study at 50% with an accepted margin of error of 5%, we obtained a total sample of 377 individuals, with a confidence level of 95%.

Categorical variables were summarized as frequencies and percentages while continuous variables were presented using median and interquartile range (IQR), since the variables were non-normally distributed. Normality was assessed both graphically and using the Shapiro–Wilks test.

We carried out multinomial logistic regression to investigate the association between the socioeconomic characteristics, the other variables investigated in the questionnaire and the outcome. The studied outcome was the intention of parents to immunize children categorized into three groups: (1) those who declared a positive intention to vaccinate or those who had already vaccinated their children; (2) those who were uncertain about their children’s vaccination; and (3) those who would not have their children vaccinated. We stratified the model by children’s age (<12 years vs ≥12 years). To define this category, we considered the age of the eldest child (i.e., if a family had a child <12 years and one ≥12 years, it was categorized as ≥12 years).

We carried out multiple imputations with chained equations [21] to generate values for missing data such as region, sex, person completing the survey, respondent parent’s education, number of household members, underlying disease in parents and/or other household members, respondent’s flu vaccine, respondent’s COVID-19 vaccination, history of COVID-19 in parents or household members, perceived risk of getting COVID-19, hospitalization and COVID-19 transmission for children. The percentage of missing data was low (4.75%). All variables included in the models as predictors of outcome were used to predict missing values [21,22]. Data were assumed to be “missing at random” [22]. Twenty-five datasets were imputed. Outcome was not imputed.

Data analysis was performed with STATA 14.1 SE (Stata Corporation, College Station, TX, USA).

## 3. Results

A total of 2808 persons accessed the online questionnaire via the provided link. Of these, 425 were excluded as they were not eligible as they did not have children, 610 accessed the questionnaire link but did not give consent to participating in the study and therefore were not included, and 77 did not complete the questionnaire. A total of 1696 participants were thus included in the study. Of these, 491 were parents of at least one child ≥ 12 years, while the remaining 1205 (71.0%) only had <12 year-old children.

Table 1 shows the general characteristics of the studied population.

A total of 1381 respondents (81.6%) were mothers and 312 (18.4%) were fathers, with a median age of 42 years (IQR 37–47). Most respondents were from Central Italy (72.9%), followed by Northern Italy (20.6%) and Southern Italy (6.5%). Most parents (1223/1696, 72.1%) had a university degree or a higher level of education, one quarter of participants had a high school certificate (421/1696, 25.0%) and only 2.9% (48/1696) had a primary or secondary school certificate.

Most families had three (717/1696, 43.1%) or four (645/1696, 38.7%) household members. In parallel, most families (50.7%) had one child or two children (40%). Seventy-four (15.4%) families had at least one person with an underlying disease.

Among the responding parents, 30% had received the influenza vaccination for the 2020/2021 season. A total of 278 (16.8%) had been affected by COVID-19 in the previous months. Most of the responding parents (1110/1696, 67.5%) had received the COVID-19 vaccine or had scheduled their vaccination, 17 (1%) intended to get the vaccine but had not received it yet as they had recently had COVID-19. Almost one fourth of the respondents (403/1696, 24.5%) stated they did not want to receive the vaccine, while 7.0% (115/1696) were undecided.

**Figure 1:**
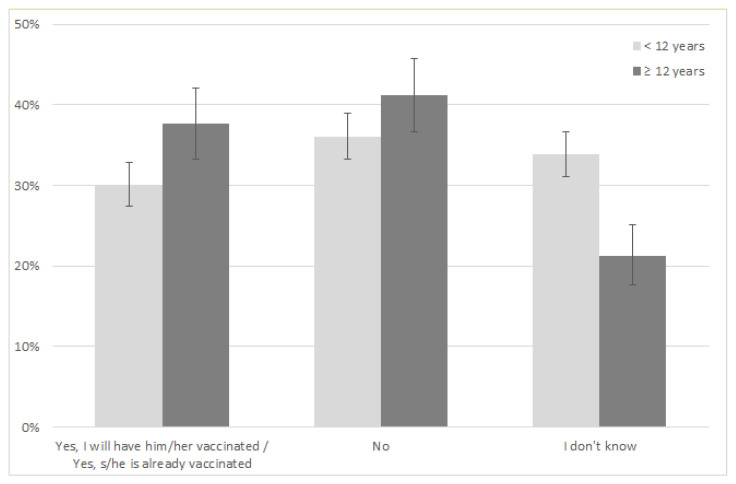
Intention to vaccinate children by target age group.

Figure 1 shows the intention to vaccinate by target age group.

As for children ≥ 12 years, most parents stated they would not vaccinate their children (41.2%, 95% CI 36.7–45.8). The proportion of parents with a positive vaccination intention (i.e., parents that already vaccinated or declared that they would vaccinate their children) was 37.61% (95% CI 33.2–42.1). Undecided parents represented 21.22% of the sample (95% CI 17.63–25.17).

For children < 12 years, most parents declared a negative vaccine intention (36.1%, 95% CI 33.3–39.0). In this age group, the proportion of parents of children already vaccinated was extremely low (0.26%), as the vaccine has been offered only to children <12 years with underlying diseases, with a very high risk of COVID-19. Overall, the proportion of parents of children <12 years who declared a positive vaccine intention was 30% (95% CI 27.4–32.8). Undecided parents represented 33.83% of the sample (95% CI 31.1–36.6).

Table 2 shows perception of COVID-19 and COVID-19 vaccination by the study population. Regarding perception of COVID-19 risks for children, 941 participants (58.1%) believed that persons < 18 years of age had a similar or higher risk of getting COVID-19 compared to adults (<12 years 58.1%, ≥12 years 59.2%).

A total of 363 (22.5%) persons stated that the available vaccines were almost completely ineffective. A very high proportion of parents stated that vaccines could cause serious side effects in children (1538/1696, 90.7%; <12 years 90.0%, ≥12 years 91.5%) and 21.9% (372/1696) believed that vaccines put children’s lives at risk (<12 years 18.5%, ≥12 years 30.4%).

Table 3 shows reasons for positive or negative intention to vaccinate, by target age group.

Among parents of children < 12 years, those with a positive intention to vaccinate (i.e., those that declared they would vaccinate or that had already vaccinated their child) provided as the two main reasons for their positive vaccination intention that the vaccine would protect their child from COVID-19 and its complications (73%) and will contribute to herd immunity (71.2%). More than a half (57.3%) stated that the vaccine was the quickest way to have children back to normal life, and for slightly less than a half (48.8%) the child’s vaccine would protect the rest of the family. In the same age group, among those with a negative intention to vaccinate and the undecided, the most frequently reported reason for not vaccinating or being hesitant was that the vaccines had not been studied enough in children (73.4%). Fear of side effects was reported by 46.3%. Only 8% of respondents stated they did not trust vaccines in general.

Among parents of children ≥ 12 years, those with a positive intention to vaccinate (i.e., those that declared they would vaccinate or that had already vaccinated their child) provided as the main reasons for their positive vaccination intention that the vaccine will contribute to herd immunity (71.5%). A total of 64.8% stated that the vaccine was the quickest way to have children back to normal life, and 59.8% stated that the vaccine would protect their child from COVID-19 and its complications. In the same age group, one third of those with a negative intention to vaccinate and the undecided reported that the vaccines had not been studied though in children (66.3%). Fear of side effects was reported by 53.9%. Almost 30% reported that the COVID-19 vaccine had a low efficacy, and 13.5% of respondents stated they did not trust vaccines in general.

According to the multinomial analysis (reported on Table 4) for parents of children < 12 years (an age group for which at the time of the present study the vaccine was not yet available), factors with a statistically significant association with a negative intention to vaccinate were: not having received the influenza vaccine, believing that children had a lower or absent risk compared to adults of getting COVID-19, being hospitalized for COVID-19 and transmitting the virus to others, believing that the vaccine efficacy was lower than 70% and that vaccines do cause major side effects, believing that vaccines are dangerous. For the same age group, factors significantly associated with being undecided were: not having received the influenza vaccine, believing that children had a lower or absent risk compared to adults of getting COVID-19 and being hospitalized by COVID-19, believing that vaccine efficacy was lower than 70% and that vaccines do cause major side effects, believing that vaccines are dangerous. Interestingly, hesitant parents were more frequently vaccinated compared to parents with a positive vaccine intention.

For parents of children ≥ 12 years, fathers significantly more frequently stated they refused to vaccinate their children compared to mothers. Other factors significantly associated with a negative intention to vaccinate were believing that children had a low risk of transmitting the virus to others compared to adults, believing that the vaccine efficacy was lower than 70% and believing that vaccines were dangerous. For the same age group, factors significantly associated with being undecided were: believing that children had a lower or absent risk of being hospitalized by COVID-19 compared to adults, believing that the vaccine efficacy was lower than 70% and believing that vaccines are dangerous.

## 4. Discussion

After several disease waves and a very high toll in terms of deaths and disabilities, we now have efficacious vaccines available to combat the global spread of COVID-19. Most countries, after a vaccination campaign targeting the elderly, patients with chronic diseases and, subsequently, adults of other age ranges, have started focusing on younger persons to slow down the virus circulation. The rapid introduction of a novel vaccine for children should be accompanied by a comprehensive communication strategy, which may not be able to be fully accomplished during a pandemic, due to time and resource constraints. Studying the relationship between concerns and vaccine behavior of target populations and measuring their level of confidence toward SARS-CoV-2 vaccines is crucial to tailor information strategies and to prevent low immunization coverage.

Our survey shows alarming results regarding the intention of families to immunize their children, which is lowest in parents of children below 12 years of age. When reviewing the current literature on this topic we found similar results in several other studies performed in other countries at different times, including the US, Germany, Australia and China [23,24,25,26]. When comparing our results to other Italian data, a previous study by Fedele et al. showed a proportion of parents refusing the COVID-19 vaccine for children similar to ours (34.5%), a lower proportion of parents favorable to the vaccine (17.2%) and a higher undecided population (48.3%). The difference might be due to the fact that the mentioned survey was conducted in November 2020 (before the vaccine for adults was available). Overall, this comparison shows that, despite the two study populations being different in terms of geographic setting, Italian parents with a negative child vaccine intention (slightly more than 1/3 of the total population) remained more or less stable between November 2020 and August 2021, while, in the same time period, those with a positive vaccine intention have increased and the undecided have decreased. In view of this result, we can hypothesize that the roll-out of the COVID-19 vaccination in adults has improved general confidence in this vaccine in part of the hesitant population, but a large proportion of parents have not changed their negative vaccine intention. On the other hand, surprisingly, our data are quite different from the Johns Hopkins Center for Communication Programs data, which suggest a much higher proportion of parents that would vaccinate their children. This difference could be due to a different selection process of the study population.

On one hand, it is expected that intention to vaccinate is lower for novel vaccines compared with already available ones. On the other hand, this should prompt public health agencies to rapidly implement tailored information strategies based on current data on public information needs.

As intention to vaccinate and vaccine confidence may vary over time because of different circumstances, we conducted a survey immediately after the start of immunization campaigns for 12–18-year-old children, when SARS-CoV-2 vaccines were not yet available for those under 12. The actual monitoring of immunization in children 12–18 reports that nearly 60% of the target population in this age group received two doses of the vaccine as of September 2021 [27]. Although we may have selected participants in the survey who were hesitant or vaccine opponents, the results may also suggest that many public concerns about immunization of children may have been overcome during the mass campaign in families of children 12–18 years old, not necessarily because of targeted information campaigns.

Together with intention to vaccinate, we explored reasons for vaccine confidence and vaccine hesitancy as reported by interviewed families. In essence, there is room for improvement of families’ knowledge about the role of children in sustaining the circulation of infection, the safety of vaccines, and, most importantly, the way vaccines are developed and tested in children, which is the main reason for a negative intention to vaccinate and for vaccine hesitancy in our sample. These areas of knowledge need to be better addressed to create a solid foundation for public trust in COVID-19 vaccines.

In the multinomial variable model, we considered the uncertainty regarding children’s vaccination and the more definite refusal of children’s COVID-19 vaccines as two different outcomes, as these behaviors likely require different communication approaches.

Being against immunization of children younger than 12 or being uncertain about the vaccine for this age group were associated with three main beliefs. First, parents who did not receive the flu vaccine for the current season were more likely to be against COVID immunization for children. Uncertain parents were also more likely to have received a COVID-19 vaccine themselves. This finding suggests an association with general concerns about vaccines. Secondly, parents who believe that COVID-19 vaccines may cause major side effects and have suboptimal efficacy were more likely to refuse immunization of children or being uncertain, underlining an information gap. Thirdly, parents who are against immunization of children or are uncertain perceived that COVID-19 in children is unlikely and that the disease does not cause major complications in this age group, also suggesting an area for improving information.

When looking at parents of children older than 12, only perception of a low efficacy of vaccines and concerns about safety were associated with refusing immunization. Uncertain parents were more likely to have received a COVID-19 vaccine, perceived a low efficacy of vaccines, and believed that the risk of complications in children is low. Of note, being a father and a decreasing age of parents were associated with refusing COVID-19 immunization for their children in this age group.

A perceived low risk of transmission from children to other individuals was associated with refusal of COVID-19 vaccines in children of both age groups, but not in uncertain parents.

This information may be useful for developing communication strategies to promote COVID-19 immunization with specificities. For instance, communication strategies are generally focused on families but not specifically on fathers, and there are no strategies focused on adult recipients of the COVID-19 vaccine to promote immunization to their relatives. Moreover, addressing concerns on safety and efficacy of vaccines in children should be complemented with information about the development and testing of immunization products for this age group.

This work has strengths as it was conducted in a very specific time frame which may be important to understand the relationship between the public perception and their behavior in immunization decisions and included a large population. Moreover, the data analysis was conducted to address the determinants of opposition and vaccine hesitancy separately, offering specific information for communication strategies.

This survey also has limitations. As we used a convenience sample, we may have recruited respondents in the emergency room more interested in the discussion about vaccines and amplified the negative perceptions when compared to the general population. Families in the emergency room may also have been distracted by their actual child’s health problem. Moreover, we did not investigate the cause for ER access of participants and we could not adjust the analysis for this factor. Indeed, only a small fraction of the respondents had had their child already vaccinated compared to the general population. On the other hand, we were able to properly investigate the determinants associated with vaccine behaviors in a multivariable model which adjusted for confounding for the several variables we included in the model.

In conclusion, although many of the public concerns may be temporary and may not fully predict vaccine behaviors, tools for constant monitoring of information needs of the population at large during immunization strategies represent a necessary means to support vaccine confidence and uptake, reinforce trust in immunization and sustain vaccination strategies in the long term. As, based on the multivariable analysis results, fathers seem to be more frequently hesitant compared to mothers, this suggests the opportunity to assess the potential effectiveness of vaccine promotion campaigns tailored to fathers for the >12 years group. Moreover, vaccine promotion campaigns may take advantage of contacts at the time of adult immunization to promote COVID-19 vaccine for children. Moreover, a wider spread of knowledge regarding COVID-19 development process and safety should be pursued.

The balance between the perceived epidemiology of vaccine-preventable diseases and perceived vaccine efficacy and safety are of paramount importance for the success of immunization strategies and require efforts to address the concerns of the public. Misconceptions and perception of high risks associated with vaccination in children may be a cause for concern in parents of child candidates to receive a SARS-CoV-2 vaccine, despite the benefits clearly outweighing the risks even in young children [28]. Parents’ perceptions on vaccine safety and efficacy may include other factors such as accessibility to vaccine facilities, leading to vaccine delay and hesitancy [29]. For this reason a systematic and continuous monitoring of factors affecting vaccine uptake is of paramount importance [30,31].

## Figures and Tables

**Table 1:** Characteristics of the study population.

	Age Group					
	<12 y (*n* = 1205)		≥12 y (*n* = 491)		Total (*n* = 1696)	
	*n*	%	*n*	%	*n*	%
Parent completing the survey						
Father	198	16.4%	114	23.3%	312	18.4%
Mother	1006	83.6%	375	76.7%	1381	81.6%
Respondent’s age (years)						
Median (IQR)	40 (36–45)		48 (44–51)		42 (37–47)	
Geographic area						
Northern Italy	211	17.7%	134	27.6%	345	20.6%
Central Italy	898	75.3%	325	66.9%	1223	72.9%
Southern Italy	83	7.0%	27	5.5%	110	6.5%
Respondent’s education						
Primary or secondary school certificate	25	2.1%	23	4.7%	48	2.9%
High school certificate	271	22.7%	150	30.8%	421	25.0%
University degree or more	900	75.2%	314	64.5%	1214	72.1%
Number of household members						
2	64	5.4%	37	7.7%	101	6.1%
3	582	49.2%	135	28.0%	717	43.1%
4	424	35.9%	221	45.8%	645	38.7%
5 or more	112	9.5%	89	18.5%	201	12.1%
Number of children in the household						
1	657	54.5%	203	41.3%	860	50.7%
2	462	38.3%	216	44.0%	678	40.0%
3	74	6.2%	57	11.6%	131	7.7%
4 or more	12	1.0%	15	3.1%	27	1.6%
Presence of at least one person with an underlying disease in the household						
No	1542	88.2%	1045	89.7%	407	84.6%
Yes	194	11.8%	120	10.3%	74	15.4%
Respondent’s influenza vaccination for the 2020/2021 season						
No	794	67.9%	364	75.2%	1158	70.0%
Yes	376	32.1%	120	24.8%	496	30.0%
History of COVID-19 in at least one household member						
No	999	85.4%	377	77.9%	1376	83.2%
Yes	171	14.6%	107	22.1%	278	16.8%
Respondent’s COVID-19 vaccination						
No, I have had COVID-19 less than 3 months ago	10	0.9%	7	1.5%	17	1.0%
No, I won’t get the vaccine	244	21.0%	159	33.1%	403	24.5%
Vaccinated/plan to get the vaccine	826	7.1%	284	59.0%	1110	67.5%
I am not sure	84	7.2%	31	6.4%	115	7.0%

**Table 2:** Perception of COVID-19 and COVID-19 vaccination in children, by target age group.

	Age Group					
	<12 y		≥12 y		Total	
	*n*	%	*n*	%	*n*	%
Children’s risk of getting COVID-19 compared to adults						
No risk/lower/I do not know	660	57.6%	281	59.2%	941	58.1%
Similar/higher	486	42.4%	194	40.8%	680	41.9%
Children’s risk of COVID-19 hospitalization compared to adults						
No risk/lower/I do not know	998	87.2%	433	91.2%	1431	88.3%
Similar/higher	147	12.8%	42	8.8%	189	11.7%
Risk of transmitting SARS-CoV-2 by children affected with COVID-19, compared to adults						
No risk/lower/I do not know	270	23.7%	139	29.4%	409	25.3%
Similar/higher	871	76.3%	334	70.6%	1205	74.7%
Perceived effectiveness of COVID-19 vaccines						
Lower than 70%	509	44.7%	266	56.1%	775	48.1%
Higher than 70%	630	55.3%	208	43.9%	838	51.9%
Risk of serious side effects caused by COVID-19 vaccines in children						
No	121	10.0%	37	7.5%	158	9.3%
Yes	1084	90.0%	454	92.5%	1538	90.7%
Perception that COVID-19 vaccines put children’s life at risk						
No	982	81.5%	342	69.6%	1324	78.1%
Yes	223	18.5%	149	30.4%	372	21.9%

**Table 3:** Reasons for positive or negative intention to vaccinate by target age group (multiple answers allowed).

	Age Group					
	<12 y		≥12 y		Total	
	*n*	%	*n*	%	*n*	%
Reasons for positive intention to vaccinate:						
The vaccine will protect my child from COVID-19 and its complications	251	73.0%	107	59.8%	358	68.5%
The vaccine will contribute to COVID-19 herd immunity	245	71.2%	128	71.5%	373	71.3%
The vaccine is the quickest way to have my child back to normal life	197	57.3%	116	64.8%	313	59.9%
Children’s vaccine will contribute to preventing COVID-19 in the rest of the family	168	48.8%	83	46.4%	251	48.0%
I unconditionally trust vaccines	46	13.4%	8	4.5%	54	10.3%
My child has a chronic disease	4	1.2%	8	4.5%	12	2.3%
Reasons for negative intention to vaccinate:						
COVID-19 vaccines have not been studied enough in children	587	73.4%	197	66.3%	784	71.5%
Fear of side effects	370	46.3%	160	53.9%	530	48.3%
The risk of having COVID-19 complications in children is low	309	38.6%	153	51.5%	462	42.1%
The risk of getting COVID-19 in children is low	199	24.9%	104	35.0%	303	27.6%
The COVID-19 vaccine has a low efficacy	142	17.8%	87	29.3%	229	20.9%
I do not trust vaccines in general	64	8.0%	40	13.5%	104	9.5%

**Table 4:** (A) Factors associated with a negative and undecided intention to vaccinate in children < 12 years. (B) Factors associated with a negative and undecided intention to vaccinate in children ≥ 12 years.

(**A**)						
	**Intention of Parents to Immunize Children** **(Group Age: <12 Years)**					
	**No**			**Undecided**		
	**Coef.**	***p***	**95% CI**	**Coef.**	***p***	**95% CI**
Parent completing the survey (Ref: Father)						
Mother	1.26	0.489	0.66–2.39	1.25	0.362	0.77–2.03
Respondent’s age	1.00	0.952	0.96–1.03	1.01	0.664	0.98–1.03
Area/region (Ref: Northern Italy)						
Central Italy	0.72	0.365	0.35–1.47	1.02	0.959	0.54–1.92
Southern Italy	1.39	0.545	0.48–4.07	1.49	0.379	0.61–3.65
Respondent’s education (Ref: Primary or secondary school certificate)						
High school certificate	1.25	0.757	0.31–5.06	1.30	0.681	0.37–4.50
University degree or more	1.16	0.827	0.30–4.48	1.12	0.852	0.34–3.71
Number of household members	0.90	0.634	0.58–1.39	1.10	0.580	0.78–1.57
Number of children (Ref: 1 child)						
2	1.24	0.489	0.67–2.28	1.30	0.359	0.77–2.06
3 or more	1.09	0.886	0.34–3.55	0.95	0.908	0.38–2.38
Underlying disease in at least one household member (Ref: No)						
Yes	0.55	0.130	0.25–1.20	1.40	0.176	0.86–2.29
Respondent’s and/or other household member’s history of COVID-19 (Ref: No)						
Yes	1.33	0.331	0.75–2.37	0.88	0.617	0.53–1.45
Respondent’s COVID-19 vaccination (Ref: No)						
Yes	1.01	0.976	0.69–1.46	1.59	0.006	1.14–2.22
Respondent’s flu vaccination 2020/21 season (Ref: No)						
Yes	0.29	<0.001	0.18–0.45	0.57	0.001	0.41–0.79
Perceived risk of getting COVID-19 in children (Ref: No risk/lower/I do not know)						
Similar/higher	0.45	<0.001	0.29–0.69	0.65	0.012	0.46–0.91
Perceived risk of COVID-19 hospitalization in children (Ref: No risk/lower/I do not know)						
Similar/higher	0.14	<0.001	0.07–0.30	0.38	<0.001	0.24–0.60
Perceived risk of children affected by COVID-19 transmitting the virus compared to adults (Ref: No risk/lower/I do not know)						
Similar/higher	0.47	0.015	0.30–0.86	1.43	0.239	0.79–2.60
Perceived effectiveness of COVID-19 vaccines (Ref: Lower than 70%)						
Higher than 70%	0.22	<0.001	0.14–0.34	0.45	<0.001	0.31–0.67
Vaccines do not cause major side effects (Ref: No)						
Yes	0.15	<0.001	0.06–0.35	0.33	<0.001	0.21–0.52
Vaccines are dangerous (Ref: No)						
Yes	60.96	<0.001	7.76–479.03	4.59	0.158	0.56–38.18
(**B**)						
	**Intention of Parents to Immunize Children** **(Group Age: ≥12 Years)**					
	**No**			**Undecided**		
	**Coef.**	***p***	**95% CI**	**Coef.**	***p***	**95% CI**
Parent completing the survey (Ref: Father)						
Mother	0.33	0.016	0.13–0.81	0.66	0.286	0.30–1.42
Respondent’s age	0.91	0.025	0.84–0.99	0.95	0.114	0.89–1.01
Area/region (Ref: Northern Italy)						
Central Italy	0.59	0.378	0.18–1.91	4.60	0.024	1.22–17.37
Southern Italy	0.90	0.926	0.09–9.06	2.86	0.388	0.26–31.06
Respondent’s education (Ref: Primary or secondary school certificate)						
High school certificate	2.25	0.433	0.30–17.18	3.03	0.189	0.58–15.87
University degree or more	5.42	0.102	0.72–40.95	2.74	0.234	0.52–14.37
Number of household members	0.93	0.771	0.55–1.55	0.74	0.162	0.49–1.13
Number of children (Ref: 1 child)						
2	0.79	0.636	0.30–2.10	0.61	0.234	0.27–1.37
3 or more	0.49	0.380	0.10–2.39	1.11	0.871	0.32–3.90
Underlying disease in at least one household member (Ref: No)						
Yes	1.00	0.996	0.37–2.75	0.63	0.235	0.29–1.35
Respondent’s and/or other household member’s history of COVID-19 (Ref: No)						
Yes	0.95	0.909	0.39–2.30	1.66	0.162	0.82–3.39
Respondent’s COVID-19 vaccination (Ref: No)						
Yes	0.78	0.448	0.41–1.48	2.00	0.012	1.17–3.43
Respondent’s flu vaccination 2020/21 season (Ref: No)						
Yes	0.51	0.124	0.22–1.20	0.78	0.428	0.43–1.43
Perceived risk of getting COVID-19 in children (Ref: No risk/lower/I do not know)						
Similar/higher	0.59	0.196	0.27–1.31	0.57	0.069	0.31–1.04
Perceived risk of COVID-19 hospitalization in children (Ref: No risk/lower/I do not know)						
Similar/higher	0.15	0.057	0.02–1.06	0.30	0.031	0.10–0.90
Perceived risk of children affected by COVID-19 transmitting the virus compared to adults (Ref: No risk/lower/I do not know)						
Similar/higher	0.36	0.041	0.13–0.96	0.60	0.285	0.23–1.53
Perceived effectiveness of COVID-19 vaccines (Ref: Lower than 70%)						
Higher than 70%	0.12	<0.001	0.05–0.28	0.38	0.003	0.20–0.73
Vaccines do not cause major side effects (Ref: No)						
Yes	0.34	0.181	0.07–1.65	0.38	0.062	0.13–1.05
Vaccines are dangerous (Ref: No)						
Yes	45.10	<0.001	5.42–374.93	5.16	0.154	0.54–49.25

## Data Availability

The data presented in this study are available on request from the corresponding author and upon approval by the data protection officer.

## References

[1] WHO Coronavirus (COVID-19) Dashboard. https://covid19.who.int.

[2] Spiteri G., Fielding J., Diercke M., Campese C., Enouf V., Gaymard A., Bella A., Sognamiglio P., Sierra Moros M.J., Riutort A.N. (2020). First Cases of Coronavirus Disease 2019 (COVID-19) in the WHO European Region, 24 January to 21 February 2020. Eurosurveillance.

[3] Onder G., Rezza G., Brusaferro S. (2020). Case-Fatality Rate and Characteristics of Patients Dying in Relation to COVID-19 in Italy. JAMA.

[4] Antos A., Kwong M.L., Balmorez T., Villanueva A., Murakami S. (2021). Unusually High Risks of COVID-19 Mortality with Age-Related Comorbidities: An Adjusted Meta-Analysis Method to Improve the Risk Assessment of Mortality Using the Comorbid Mortality Data. Infect. Dis. Rep..

[5] Castagnoli R., Votto M., Licari A., Brambilla I., Bruno R., Perlini S., Rovida F., Baldanti F., Marseglia G.L. (2020). Severe Acute Respiratory Syndrome Coronavirus 2 (SARS-CoV-2) Infection in Children and Adolescents: A Systematic Review. JAMA Pediatr..

[6] Bhuiyan M.U., Stiboy E., Hassan M.Z., Chan M., Islam M.S., Haider N., Jaffe A., Homaira N. (2021). Epidemiology of COVID-19 Infection in Young Children under Five Years: A Systematic Review and Meta-Analysis. Vaccine.

[7] Osorio M.F., Vaca R.G. (2021). Coronavirus Disease 2019 in Children: Is It Really Mild?. Infect. Dis. Clin. Pract..

[8] Shekerdemian L.S., Mahmood N.R., Wolfe K.K., Riggs B.J., Ross C.E., McKiernan C.A., Heidemann S.M., Kleinman L.C., Sen A.I., Hall M.W. (2020). Characteristics and Outcomes of Children With Coronavirus Disease 2019 (COVID-19) Infection Admitted to US and Canadian Pediatric Intensive Care Units. JAMA Pediatr..

[9] Berardi C., Antonini M., Genie M.G., Cotugno G., Lanteri A., Melia A., Paolucci F. (2020). The COVID-19 Pandemic in Italy: Policy and Technology Impact on Health and Non-Health Outcomes. Health Policy Technol..

[10] Italian National Health Institute Epidemia COVID-19 Aggiornamento Nazionale. 21 April 2021—Ore 12:00. https://www.epicentro.iss.it/coronavirus/bollettino/Bollettino-sorveglianza-integrata-COVID-19_21-aprile-2021.pdf.

[11] Salute M. Della COVID-19 Weekly Monitoring, Report 5–11 July 2021. https://www.salute.gov.it/portale/news/p3_2_1_1_1.jsp?lingua=italiano&menu=notizie&p=dalministero&id=5562.

[12] Twohig K.A., Nyberg T., Zaidi A., Thelwall S., Sinnathamby M.A., Aliabadi S., Seaman S.R., Harris R.J., Hope R., Lopez-Bernal J. (2021). Hospital Admission and Emergency Care Attendance Risk for SARS-CoV-2 Delta (B.1.617.2) Compared with Alpha (B.1.1.7) Variants of Concern: A Cohort Study. Lancet Infect. Dis..

[13] Dattner I., Goldberg Y., Katriel G., Yaari R., Gal N., Miron Y., Ziv A., Sheffer R., Hamo Y., Huppert A. (2021). The Role of Children in the Spread of COVID-19: Using Household Data from Bnei Brak, Israel, to Estimate the Relative Susceptibility and Infectivity of Children. PLoS Comput. Biol..

[14] Li X., Xu W., Dozier M., He Y., Kirolos A., Lang Z., Song P., Theodoratou E. (2020). UNCOVER The Role of Children in the Transmission of SARS-CoV2: Updated Rapid Review. J. Glob. Health.

[15] Pfizer and BioNTech Announce Positive Topline Results from Pivotal Trial of COVID-19 Vaccine in Children 5 to 11 Years | Pfizer. https://www.pfizer.com/news/press-release/press-release-detail/pfizer-and-biontech-announce-positive-topline-results.

[16] Palamenghi L., Barello S., Boccia S., Graffigna G. (2020). Mistrust in Biomedical Research and Vaccine Hesitancy: The Forefront Challenge in the Battle against COVID-19 in Italy. Eur. J. Epidemiol..

[17] Rhodes M.E., Sundstrom B., Ritter E., McKeever B.W., McKeever R. (2020). Preparing for A COVID-19 Vaccine: A Mixed Methods Study of Vaccine Hesitant Parents. J. Health Commun..

[18] Fedele F., Aria M., Esposito V., Micillo M., Cecere G., Spano M., De Marco G. (2021). COVID-19 Vaccine Hesitancy: A Survey in a Population Highly Compliant to Common Vaccinations. Hum. Vaccines Immunother..

[19] COVID Behaviors Dashboard—Johns Hopkins Center for Communication Programs. https://covidbehaviors.org/.

[20] Sample Size Calculator by Raosoft, Inc.. http://www.raosoft.com/samplesize.html.

[21] White I.R., Royston P., Wood A.M. (2011). Multiple Imputation Using Chained Equations: Issues and Guidance for Practice. Stat. Med..

[22] Moons K.G.M., Donders R.A.R.T., Stijnen T., Harrell F.E. (2006). Using the Outcome for Imputation of Missing Predictor Values Was Preferred. J. Clin. Epidemiol..

[23] Alfieri N.L., Kusma J.D., Heard-Garris N., Davis M.M., Golbeck E., Barrera L., Macy M.L. (2021). Parental COVID-19 Vaccine Hesitancy for Children: Vulnerability in an Urban Hotspot. BMC Public Health.

[24] Zhang M.-X., Lin X.-Q., Chen Y., Tung T.-H., Zhu J.-S. (2021). Determinants of Parental Hesitancy to Vaccinate Their Children against COVID-19 in China. Expert Rev. Vaccines.

[25] Evans S., Klas A., Mikocka-Walus A., German B., Rogers G.D., Ling M., Fernando J.W., Kothe E., Westrupp E.M. (2021). “Poison” or “Protection”? A Mixed Methods Exploration of Australian Parents’ COVID-19 Vaccination Intentions. J. Psychosom. Res..

[26] Brandstetter S., Böhmer M.M., Pawellek M., Seelbach-Göbel B., Melter M., Kabesch M., Apfelbacher C. (2021). KUNO-Kids study group Parents’ Intention to Get Vaccinated and to Have Their Child Vaccinated against COVID-19: Cross-Sectional Analyses Using Data from the KUNO-Kids Health Study. Eur. J. Pediatr..

[27] Governo Italiano—Report Vaccini Anti COVID-19. https://www.governo.it/it/cscovid19/report-vaccini/.

[28] Scally G. (2021). Vaccinating Adolescents against SARS-CoV-2 in England: A Risk-Benefit Analysis. J. R. Soc. Med..

[29] Brewer N.T., Chapman G.B., Rothman A.J., Leask J., Kempe A. (2017). Increasing Vaccination: Putting Psychological Science Into Action. Psychol. Sci. Public Interest.

[30] McAteer J., Yildirim I., Chahroudi A. (2020). The VACCINES Act: Deciphering Vaccine Hesitancy in the Time of COVID-19. Clin. Infect. Dis..

[31] Solís Arce J.S., Warren S.S., Meriggi N.F., Scacco A., McMurry N., Voors M., Syunyaev G., Malik A.A., Aboutajdine S., Adeojo O. (2021). COVID-19 Vaccine Acceptance and Hesitancy in Low- and Middle-Income Countries. Nat. Med..

